# Supplementary material for: Crystal structures of human MGST2 reveal synchronized conformational changes regulating catalysis
Source: Nat Commun. 2021 Mar 19;12:1728. doi: 10.1038/s41467-021-21924-8 (PMC7979937; doi:10.1038/s41467-021-21924-8)
Supplement: Supplementary file 1 — Supplementary Information [file 41467_2021_21924_MOESM1_ESM.pdf]

Supplementary Information

Crystal structures of human MGST2 reveal synchronized conformational changes regulating catalysis

Madhuranayaki Thulasingam, Laura Orellana, Emmanuel Nji, Shabbir Ahmad, Agnes Rinaldo-Matthis and Jesper Z. Haeggström*

*Correspondence to Jesper.Haeggstrom@ki.se and madhuranayaki.thulasingam@ki.se

Supplementary Figure 1.

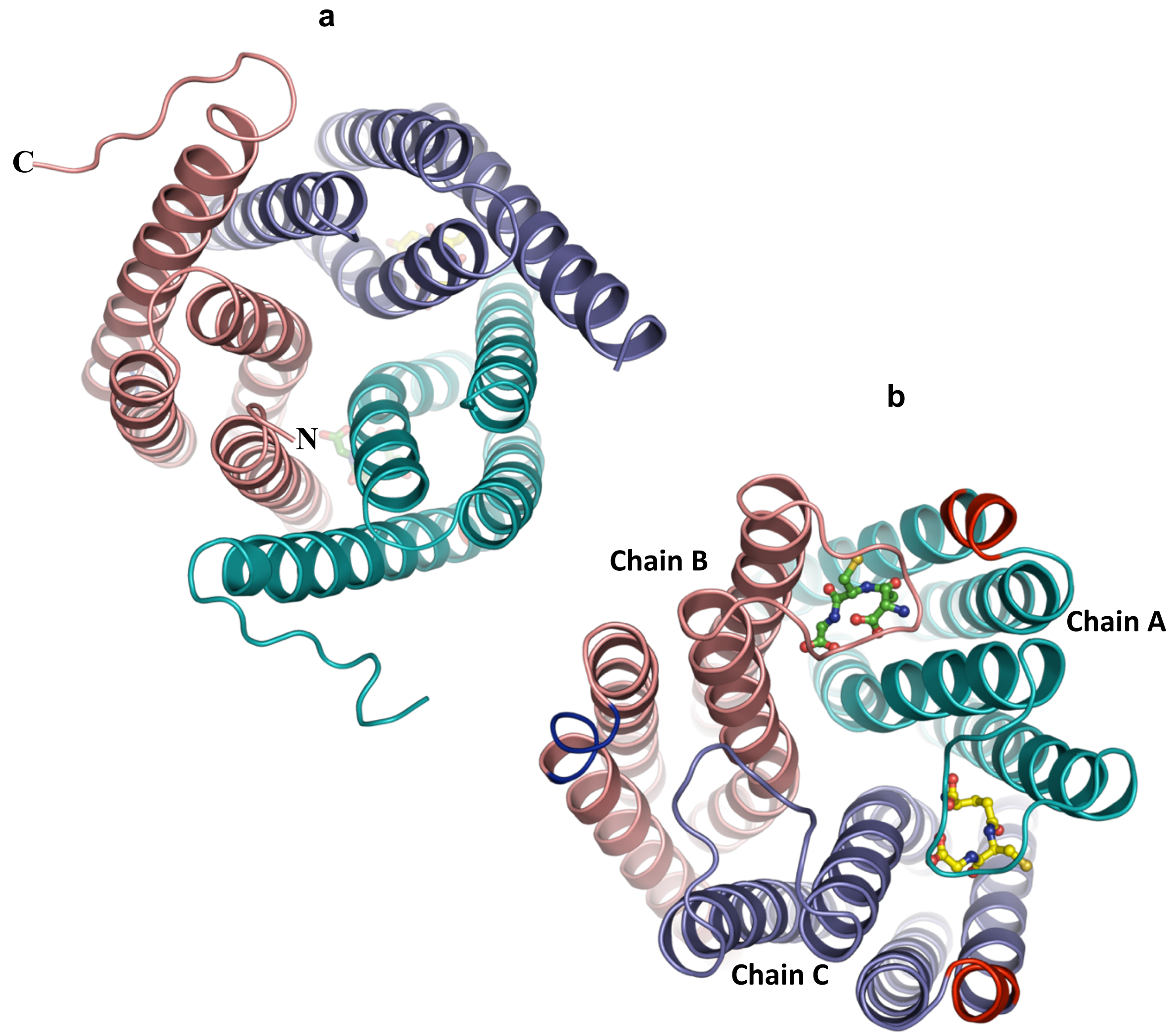

View of holo-MGST2 structure from (a) ER lumen showing the C-terminal extension of αH5 **(b)** from cytoplasm showing the loop L lid. Bound GSH molecules are shown in ball and stick rendering. 3_{10} helical motif is highlighted in red in chain A and C whereas in chain B it exists in a loop like structure highlighted in blue.

Supplementary Figure 2.

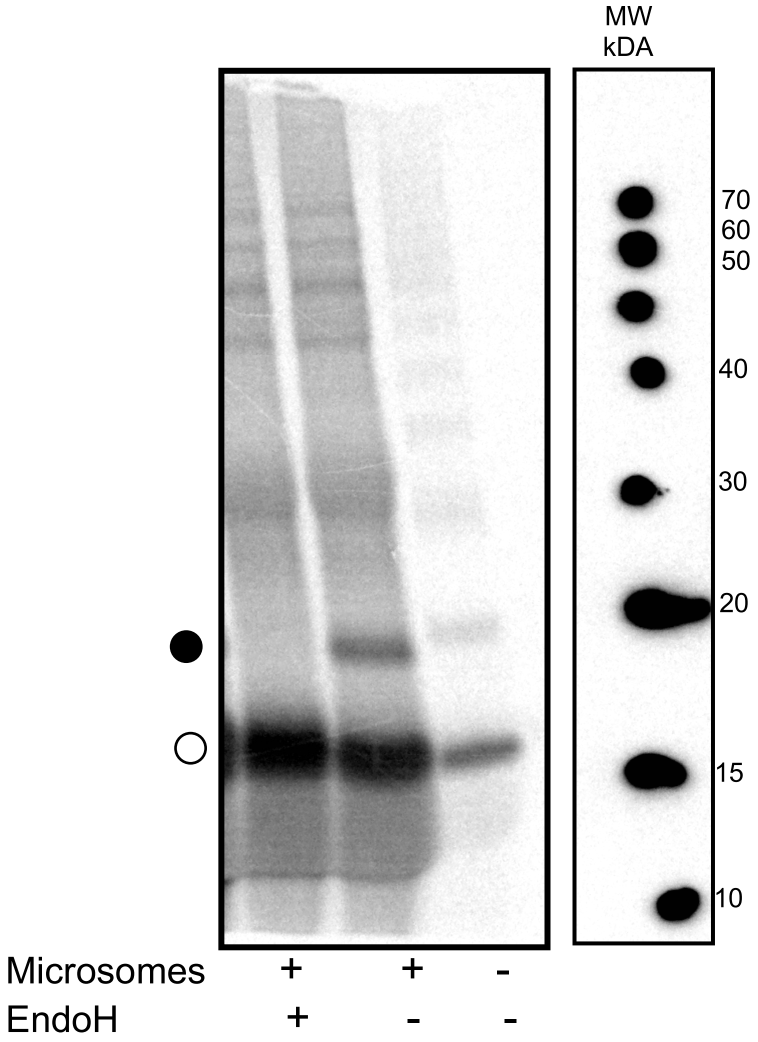

Identification of MGST2 topology by *in vitro* translation. Endo H treatment was done to confirm the glycosylation. Glycosylated and non-glycosylated bands were represented as filled and empty circles, respectively. The experiment was repeated two times with same results. Source data are provided as Source Data File.

Supplementary Figure 3.

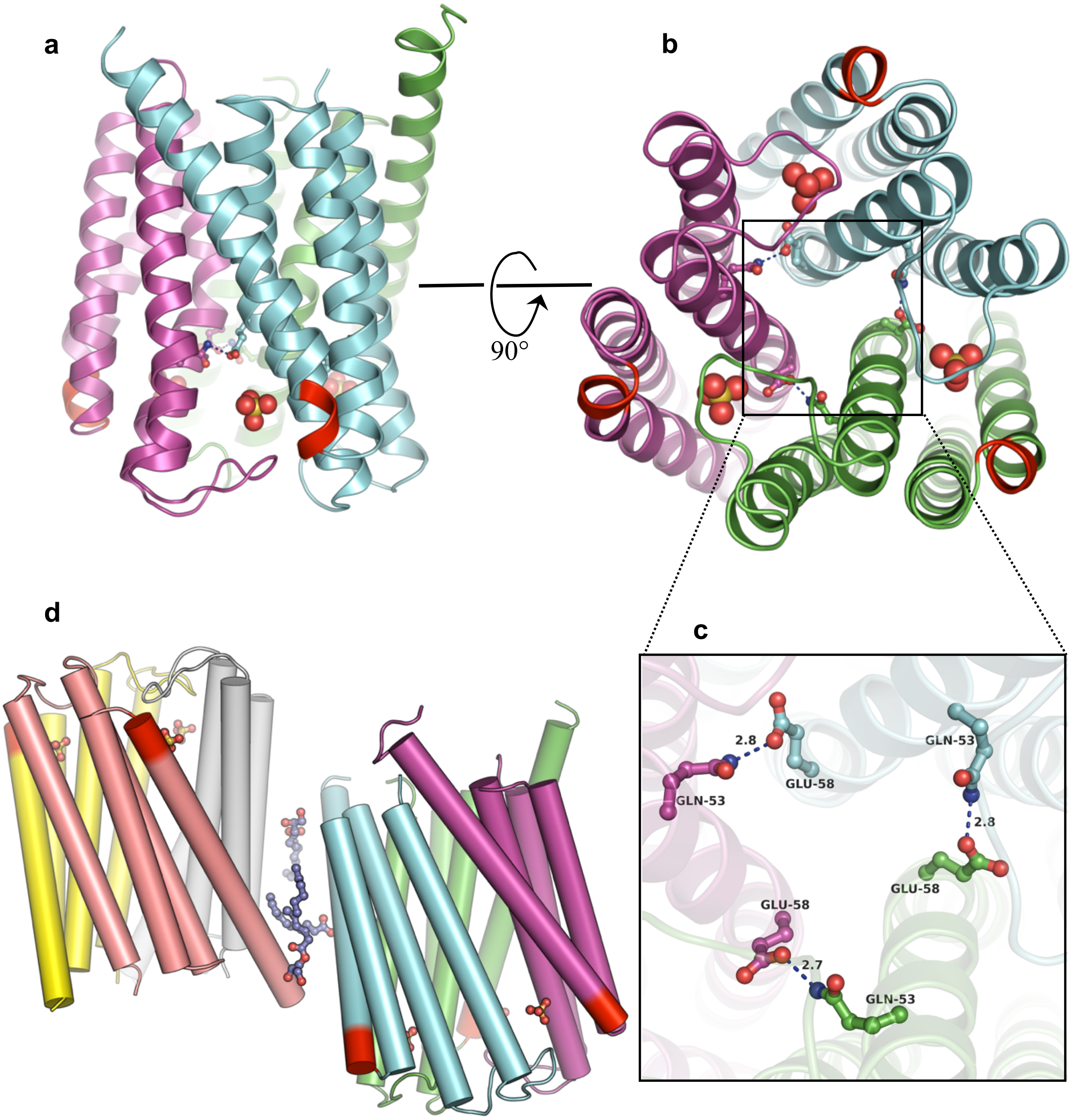

Structure of apo MGST2: (a) Cartoon rendering of MGST2 trimer with bound sulfate molecules (ball and stick) at the active site, view from the membrane plane; **(b)** view from the cytoplasm. The 3_{10} helical motif is highlighted in red; **(c)** symmetric polar inter-subunit interactions (dashed lines) established between Glu58 and Gln53 (ball and stick) located on the αH2 of each monomer; **(d)** asymmetric unit of apo MGST2 contains two molecules of trimer (Cα RMSD= 0.46Å) in cylindrical representation with lipid molecules (MAG 8.8 in purple ball and stick) at the interface.

Supplementary Figure 4.

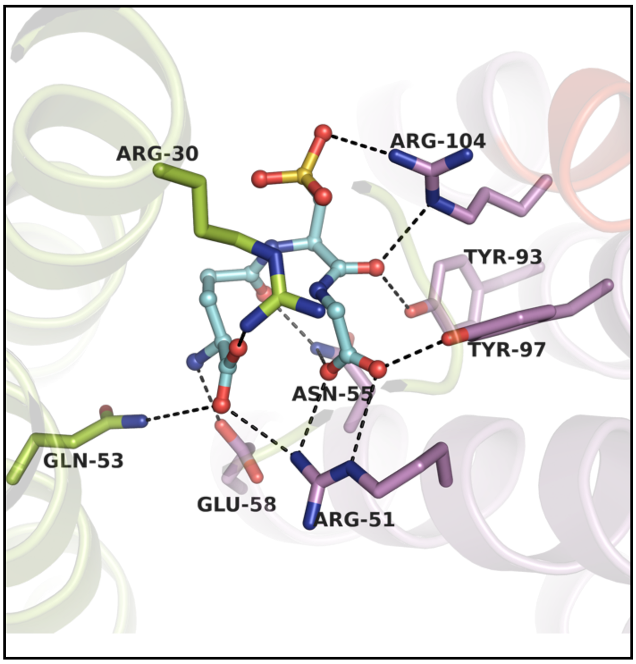

Active site MGST2-GSO_3^- complex structure: Interactions (dashed lines) of GSO_3^- molecule (cyan ball and sticks) with the surrounding active site residues (green and magenta sticks) from corresponding monomers.

Supplementary Figure 5.

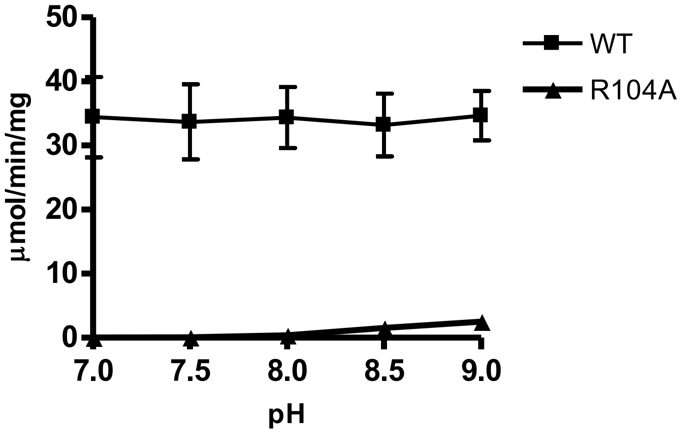

pH dependence of enzyme activity of wild type (WT) and Arg104Ala (R104A) MGST2 using 1-chloro-2,4-dinitrobenzene (CDNB) as an electrophilic substrate. The activity of the mutant enzyme is slightly rescued at pH 9 as expected. (n=3 independent activity measurements, data are presented as mean values ± SD). Source data are provided as Source Data File.

Supplementary Figure 6.

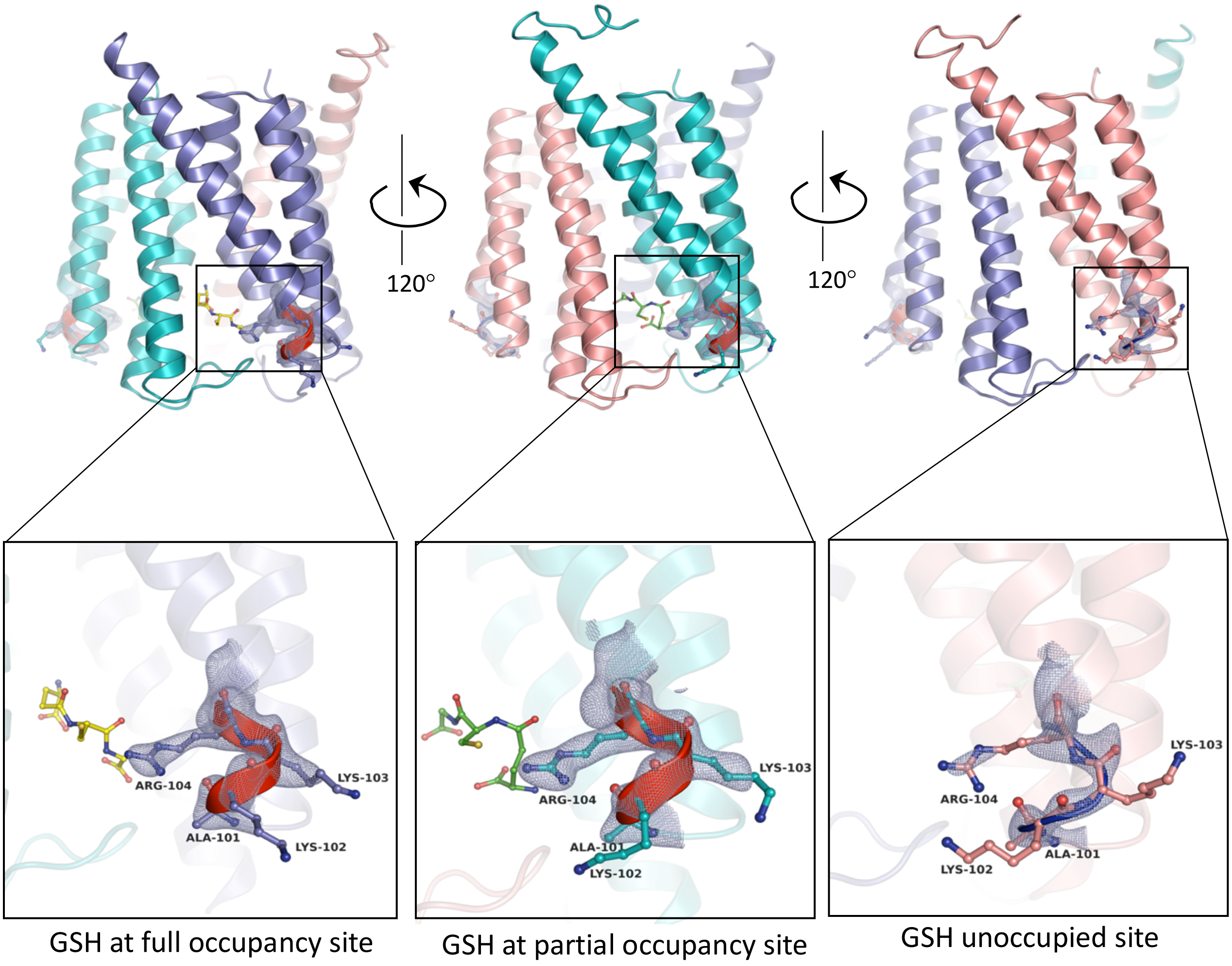

Flexibility of catalytically important Arg104 caused by the dynamics of 3_{10} helical motif: Residues correspond to the 3_{10} helical motif Ala101-Arg104 (ball and stick) are shown in 2mFo-DFc map contoured to 1.5σ from corresponding monomers. The 3_{10} helical motif of GSH bound and unbound sites are highlighted in red and blue respectively.

Supplementary Figure 7.

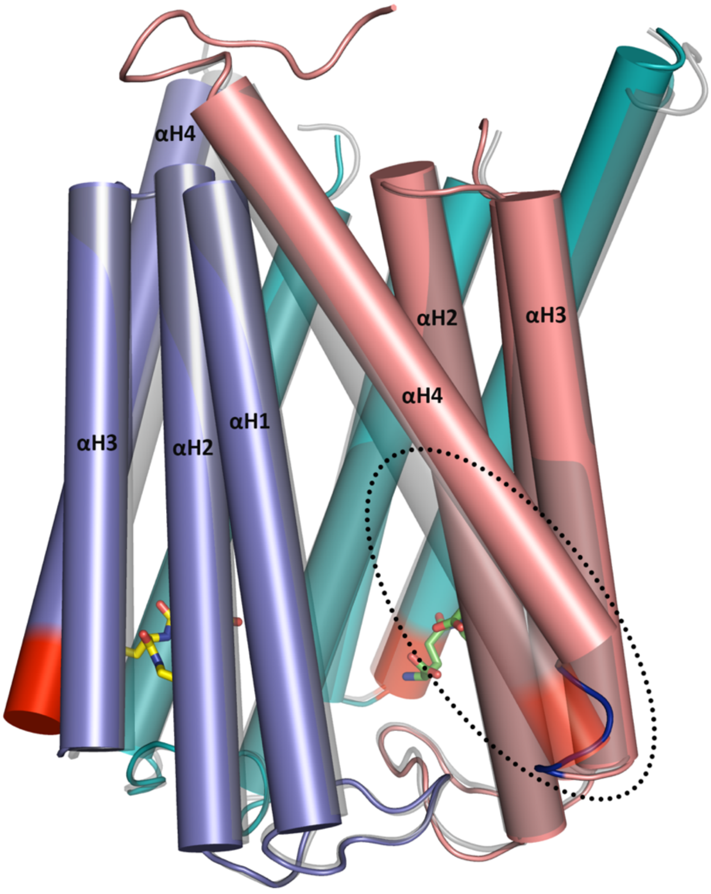

Conformational changes across the αH4: Superimposition of apo (gray translucent) and holo (opaque) MGST2 structures in cylindrical rendering showing the tilt of αH4 caused by the loop conformation of 3_{10} helical motif at the active site without GSH.

Supplementary Figure 8.

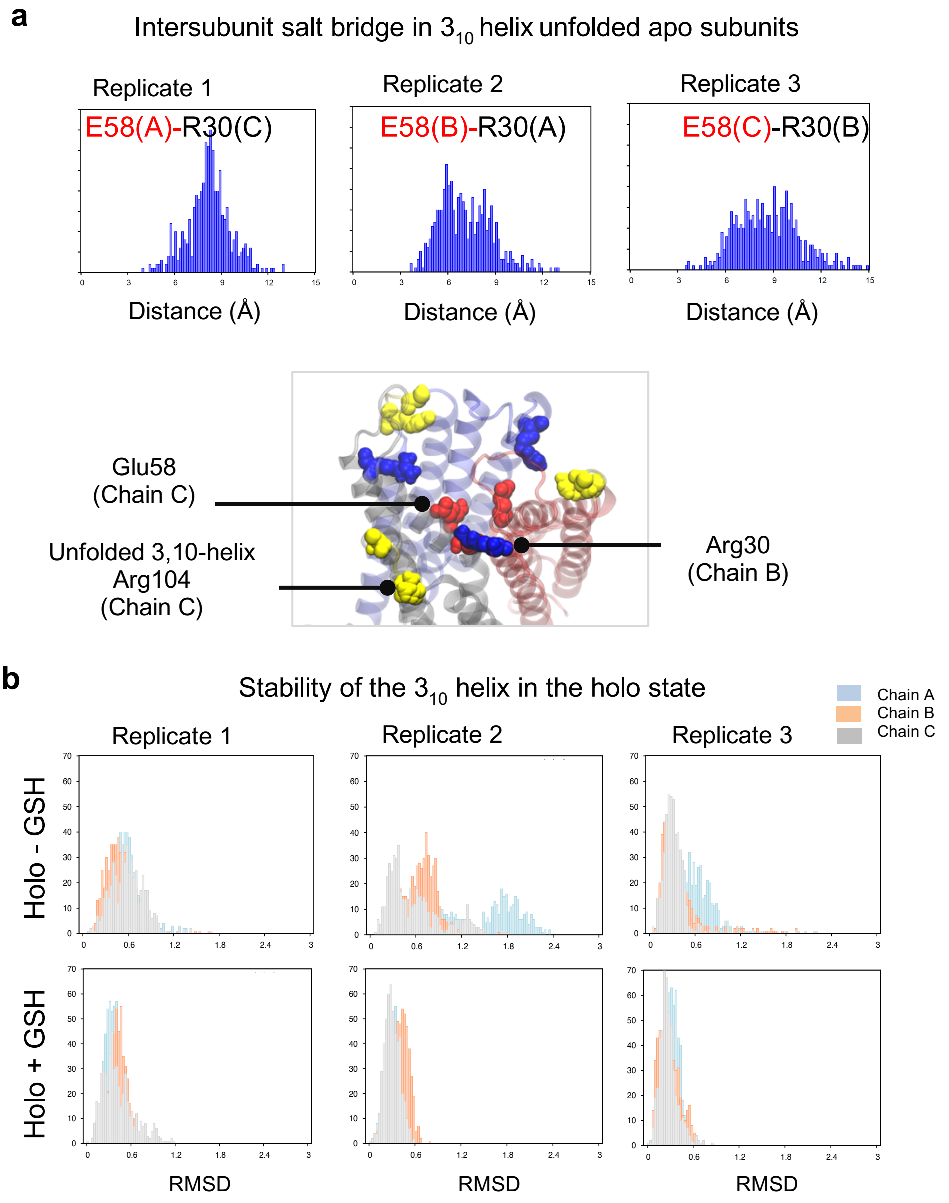

Asymmetry of inter-subunit interactions and stability of 3_{10} helical motif in MD simulations. (a) Asymmetry of intersubunit interactions in apo simulations. Glu58 of the unfolded subunit forms a transient salt bridge with Arg30 of the neighboring one. On the top, distance distribution for the Glu58-Arg30 bond, with the glutamate at the unfolded subunit highlighted in red. A snapshot for replicate 3 is shown on the bottom. (b) 3_{10} helical unfolding in simulations starting from the holo MGST2 structure**.** RMSD distribution for three 500ns MD replicate simulations of holo MGST2 in the absence (top panel) and in the presence (bottom panel) of GSH. Note how, in comparison with the apo state (Fig.5C, top panels), the 3_{10} helical motif is more stable in the holo conformation, as shown by the absence of tall peaks at higher RMSD. Binding of GSH further stabilizes the 3_{10} helical motif, resulting in narrow RMSD peaks close to the starting holo conformation.

Supplementary Figure 9.

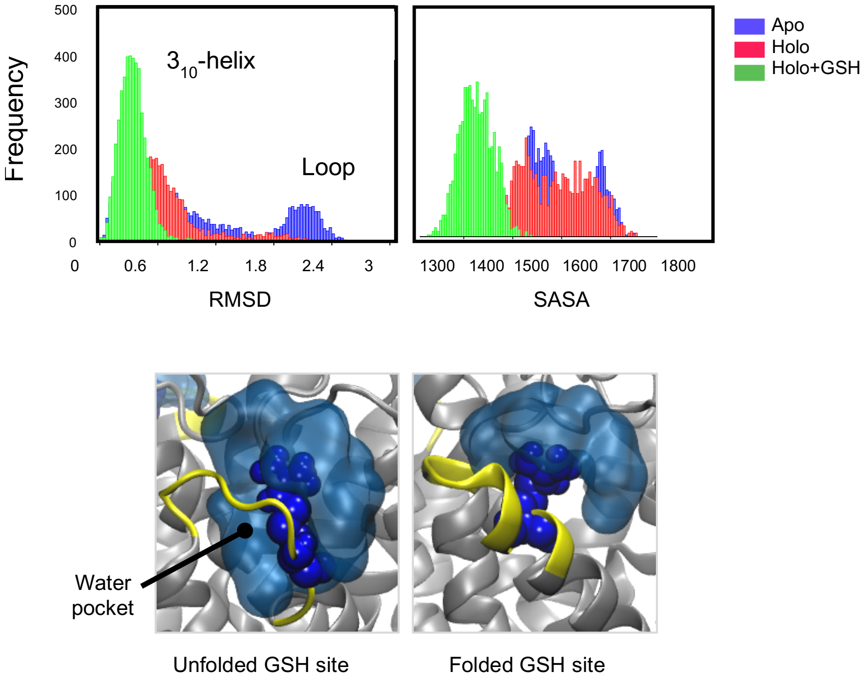

Unfolding of 3_{10} helical motif and solvent accessibility in MD simulations: Unfolding of 3_{10} helix is accompanied by an increase in solvent accessibility (top panels) at the GSH binding site (bottom panels). Note the larger water pocket (blue surface) where the 3_{10} helical motif is unfolded.

Supplementary Figure 10.

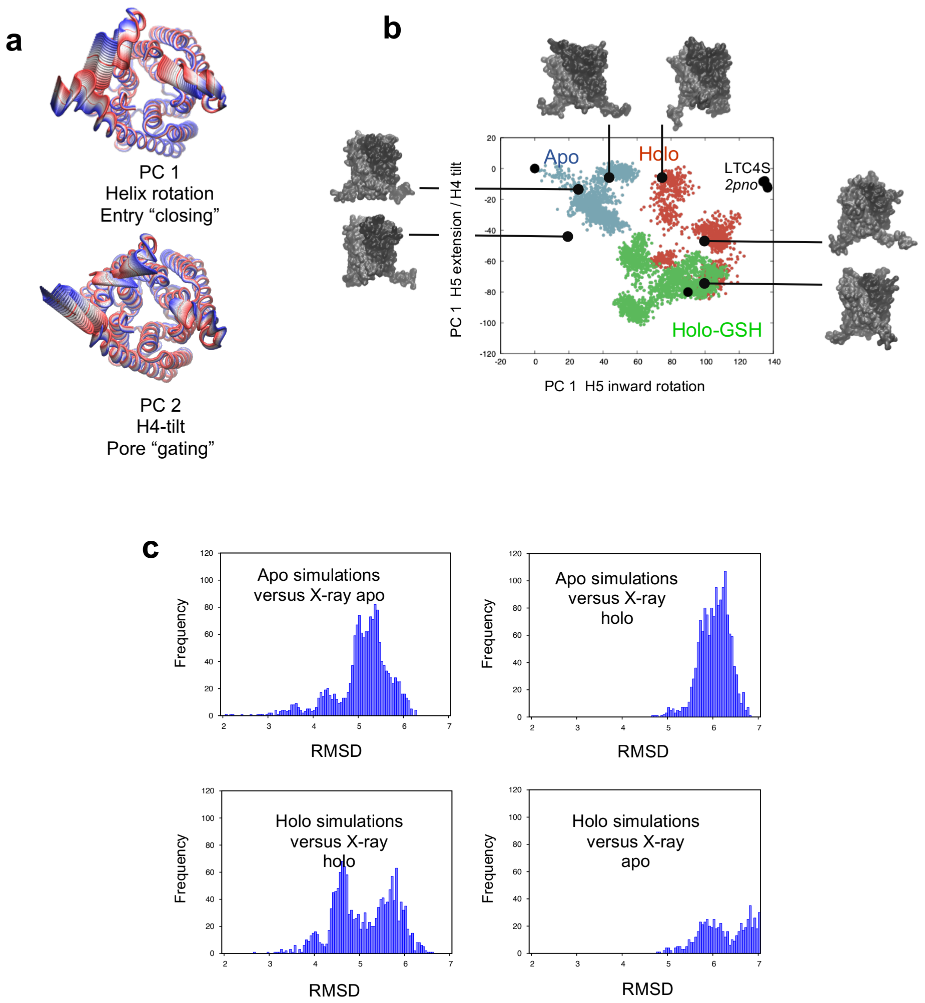

Spontaneous sampling of apo and holo states in MD: To monitor global conformational changes in simulations, we used principal components (PCs) analysis. **(a):** PCs computed from the X-ray ensemble formed by MGST2 structures and the structurally similar LTC4S (PDB ID: 2PNO). PC1&2, which capture >80% of the X-ray apo-holo change, cluster separately the two conformations. Note how MD simulations from the apo state sample half of the pathway towards the holo region and vice versa. (**b**): Conformational landscape sampled by atomistic simulations starting from the apo (*blue*) and holo state with (*green*) and without GSH (*red*), projected onto the X-ray PC-motion space. Note how unbound simulations tend to converge in the central area between apo/holo starting conformations (black circles), indicating there is spontaneous interconversion (see c). Holo simulations with GSH stay closer to the starting X-ray structure (RMSD ~ 3.9 ± 0.8 for holo, versus 5.1 ± 0.7 for holo+GSH). Lower Panel: RMSD distribution for MD simulations from the unbound apo (upper row) and holo (lower row) states. The apo and holo states approach each other within ~4.5-4.8Å.

Supplementary Figure 11.

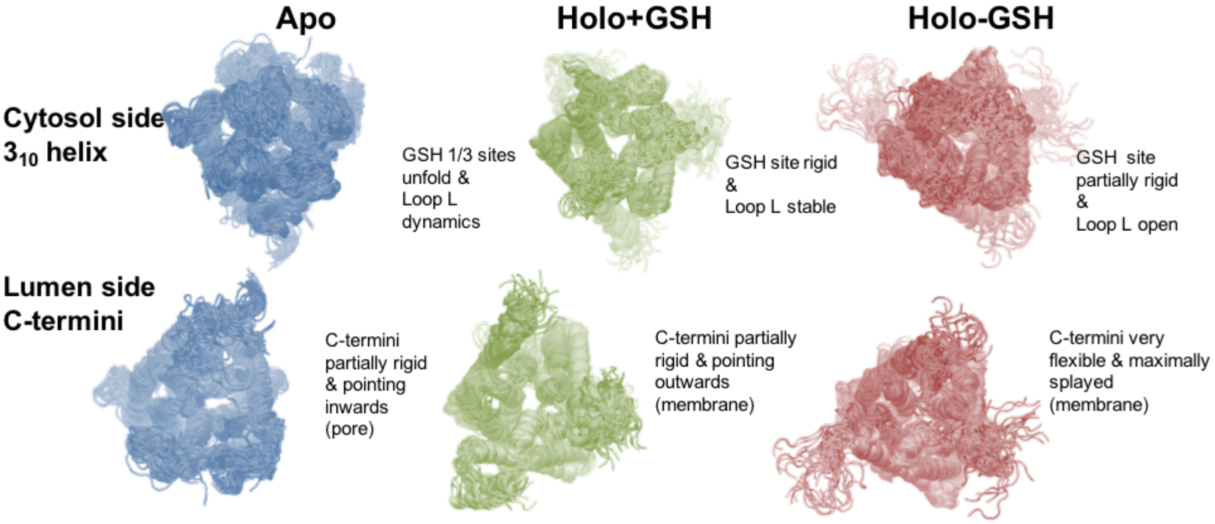

Cytosol-lumen asymmetric dynamics in MD simulations: The cytosolic side (up) displays unfolding of the 3_{10} helix and marked flexibility of the loop L in the apo state, which facilitates substrate entry, and becomes more compact and rigid upon GSH binding. Similarly, the luminal side is more flexible in unbound state, although apo and holo conformations differ markedly in the orientation of H5.

Supplementary Figure 12.

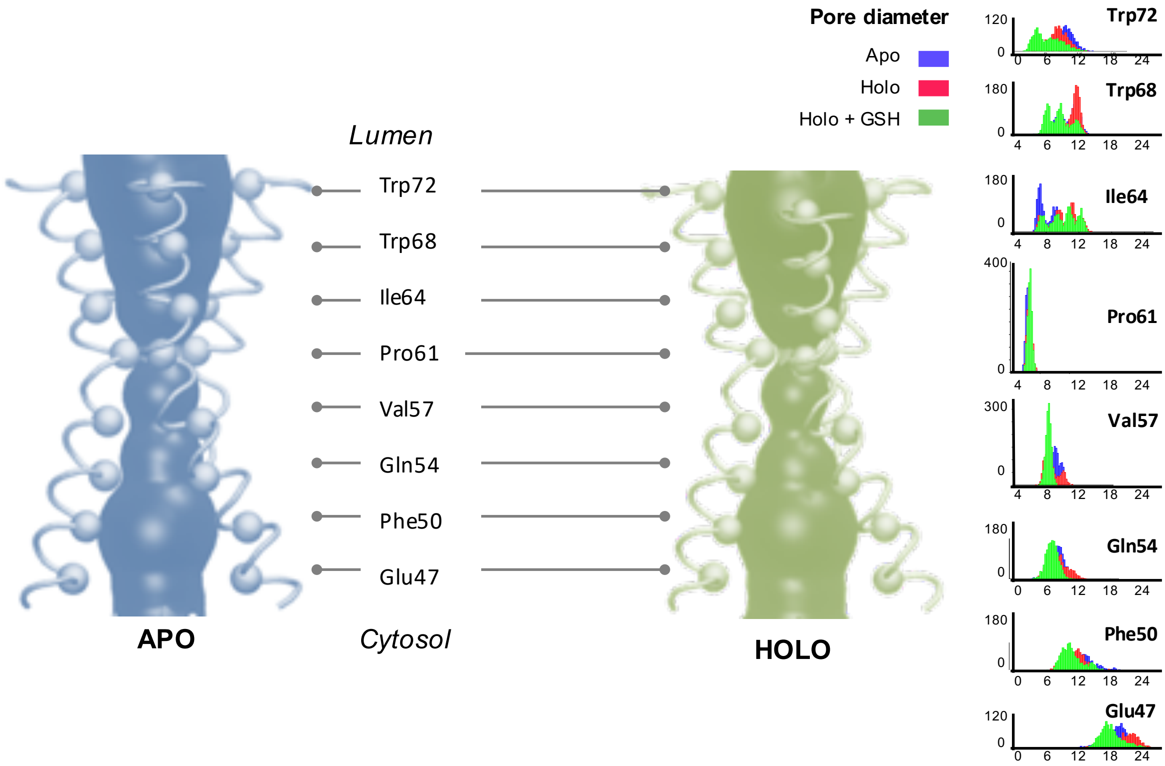

Pore diameter changes along the central MGST2 axis in MD simulations: Note the constriction at the Pro-kink and how cavity diameters are in general wider in the absence of GSH. Note also how the luminal side has very marked dynamics in the presence of GSH, manifested as different peaks for open, intermediate and closed diameters (visible for Ile64, Trp68 and less for Trp72)

Supplementary Figure 13.

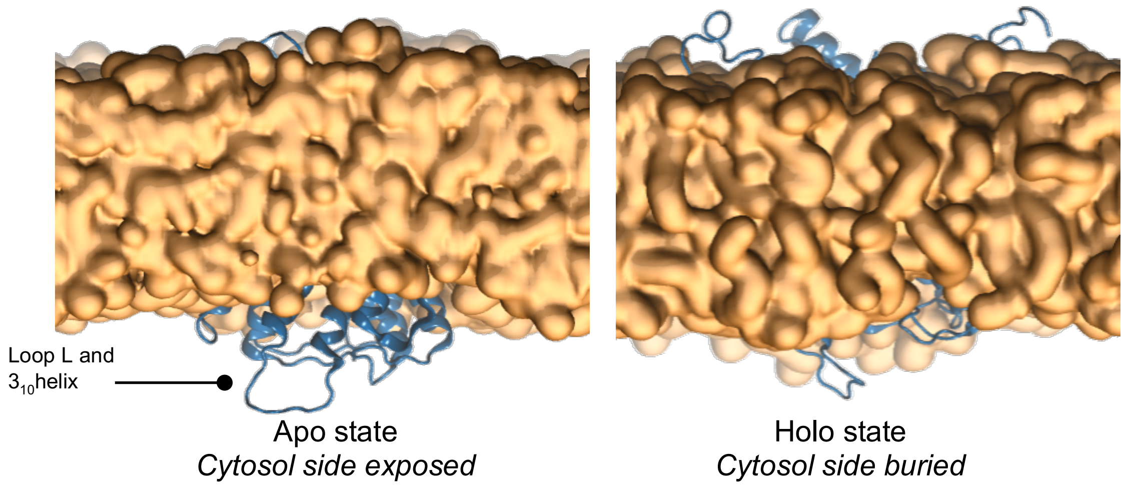

Transversal movement of MGST2 in MD simulations: Representative snapshots from apo (left) and holo (right) simulations. Dynamic loop L and 3_{10} helix in apo structure protrudes out while the flexible cytoplasmic part is stabilized and buried in the membrane when GSH is bound to the active site.

Supplementary Figure 14.

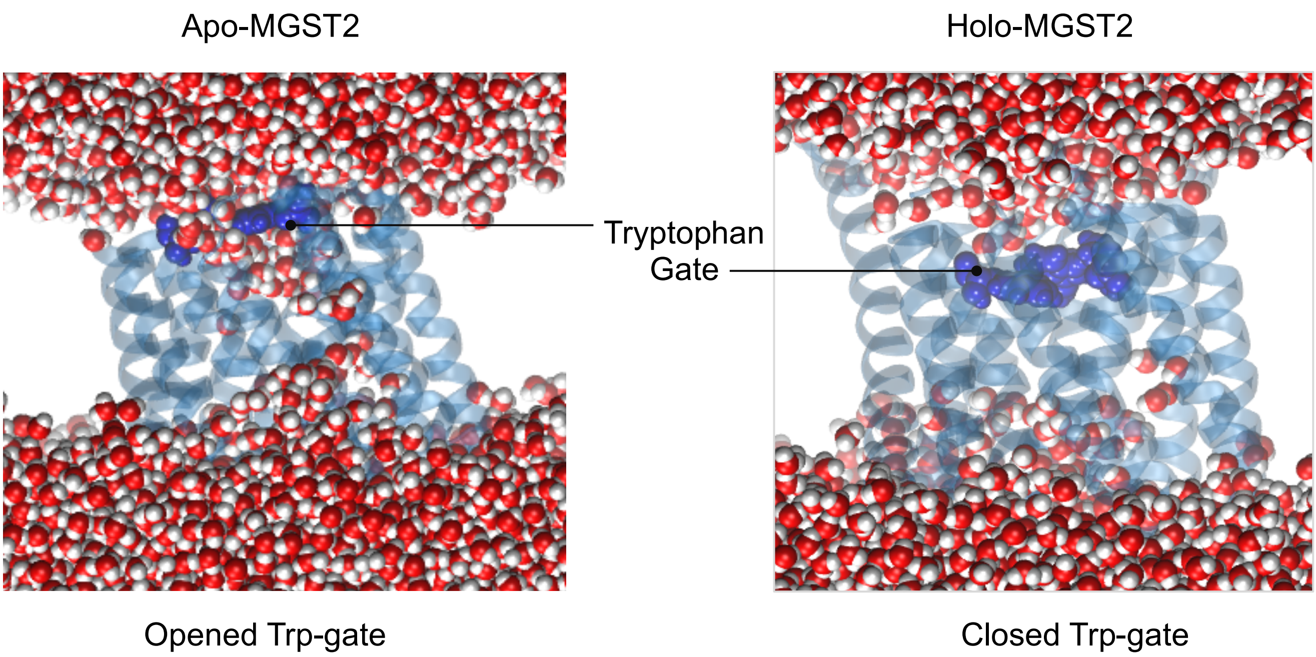

Solvent access through tryptophan gate in MD simulation: Representative snapshots from apo (left) and holo (right) simulations. Opened Trp-gate in the apo structure allows the entry of water molecules through the central channel traced from the MD simulation, whereas the Trp-gate is closed in holo-MGST2, restricting the solvent access to the active site.

Supplementary Figure 15.

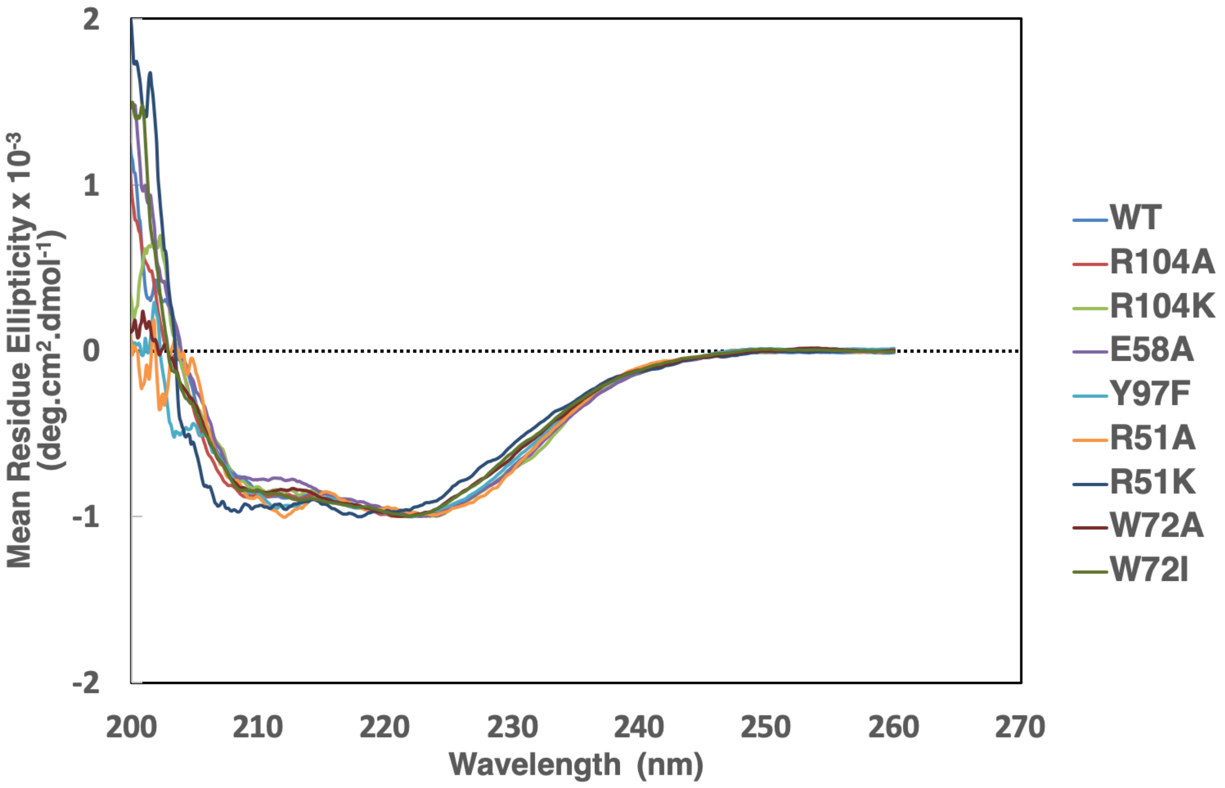

CD spectra of MGST2 wild type and mutants

Supplementary Figure 16.

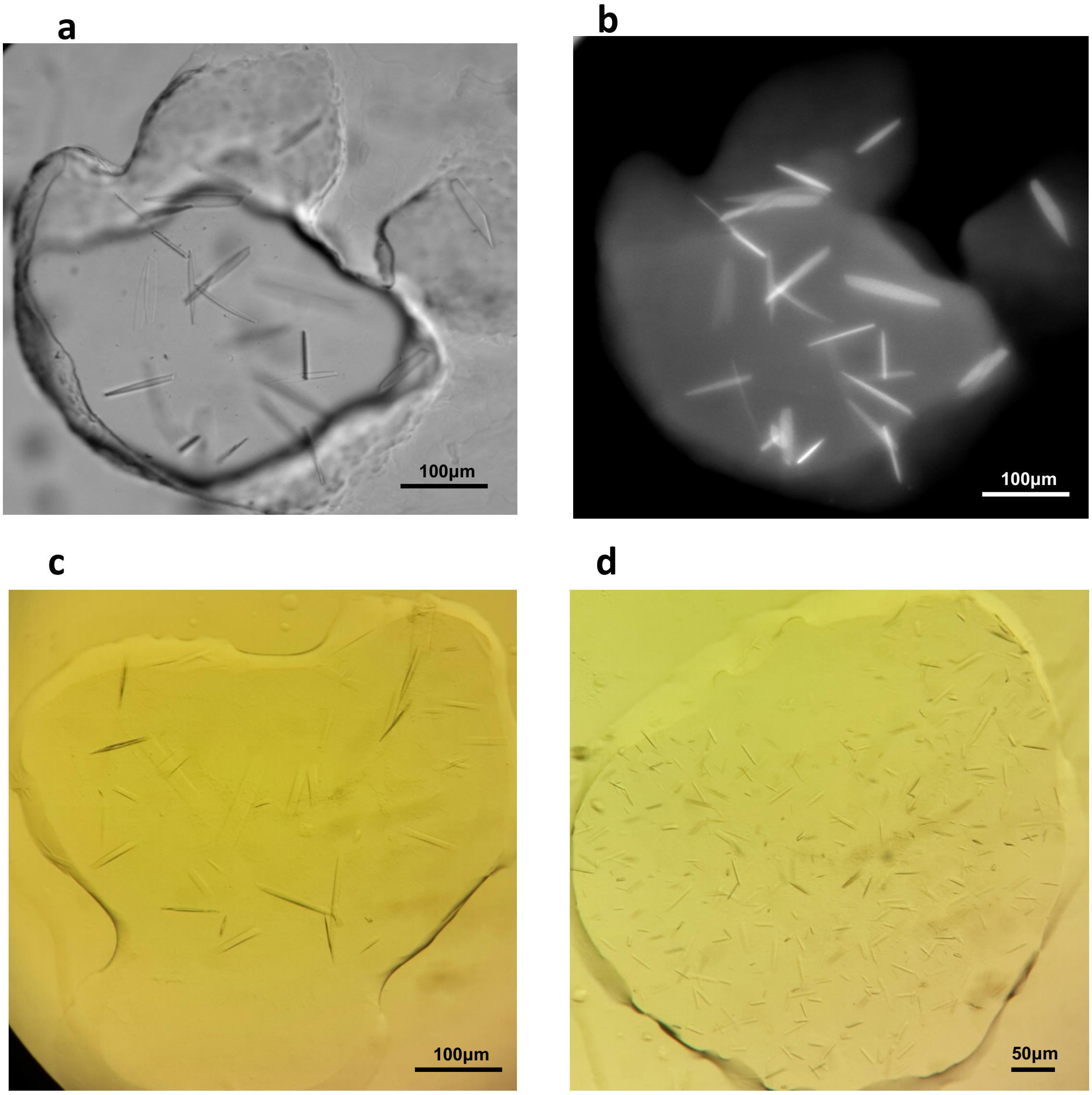

Crystals of apo-MGST2 (a) under bright field, **(b)** under UV, **(c)** MGST2-GSH and **(d)** MGST2-GSO_3^- under polarizer grown in lipidic cubic phase. Crystallization has been successfully repeated at least five times with reproducible crystals.

Supplementary Table 1.

Enzyme	k_{cat} (s^{-1})	K_M (μM)	k_{cat}/K_M (M^{-1} s^{-1})
WT MGST2	14.30±0.54	200±20	(7.2±0.4) x 10^4
R104A MGST2	0.17±0.05	1400±560	(1.5±0.3) x 10^2

Steady state kinetic parameters of wild type MGST2 and Arg104Ala (R104A) MGST2, using CDNB as an electrophilic substrate.

Supplementary Table 2.

**Primers**	**Sequence 5' to 3'**
R104A forward	GCTGCTAAAAAAGCGATCACCGGTTTCCGACTGAGTC
R104A reverse	GACTCAGTCGGAAACCGGTGATCGCTTTTTTAGCAGC
R104K forward	GCTGCTAAAAAAAAGATCACCGGTTTCCGACTGAGTC
R104K reverse	GACTCAGTCGGAAACCGGTGATCTTTTTTTTAGCAGC
R51A forward	GAGAGAGTATTTGCGGCACAACAAAAC
R51A reverse	GTTTTGTTGTGCCGCAAATACTCTCTC
R51K forward	GAGAGAGTATTTAAGGCACAACAAAAC
R51K reverse	GTTTTGTTGTGCCTTAAATACTCTCTC
Y97F forward	TACTTCTGGGGATTTTCAGAAGCTGCTAAAAAA
Y97F reverse	GTTTTTTAGCAGCTTCTGAAAATCCCCAGAAGTA
E58A forward	CAAAACTGTGTGGCGTTTTATCCTATA
E58A reverse	TATAGGATAAAACGCCACACAGTTTTG
W72A forward	GGATGGCTGGGGCGTATTTCAACCAAG
W72A reverse	CTTGGTTGAAATACGCCCCAGCCATCC
W72I forward	GGATGGCTGGGATCTATTTCAACCAAG
W72I reverse	CTTGGTTGAAATAGATCCCAGCCATCC

List of primers used in this study
