# Supplementary material for: Crystal structures of human MGST2 reveal synchronized conformational changes regulating catalysis
Source: Nat Commun. 2021 Mar 19;12:1728. doi: 10.1038/s41467-021-21924-8 (PMC7979937; doi:10.1038/s41467-021-21924-8)
Supplement: Supplementary file 6 — Description of Additional Supplementary Files [file 41467_2021_21924_MOESM6_ESM.docx]

Description of supplementary information

Title: Supplementary Movie 1.

Description: Simulation of apo-MGST2 structure shows the unfolding of 310 helix at one monomer at a time.

Title: Supplementary Movie 2.

Description: Simulation of holo-MGST2 shows the exit of GSH bound at partial occupancy from the active site.

Title: Supplementary Movie 3.

Description: Simulations of apo-MGST2 with GSH shows the entry of GSH into the active site via loop L opening
