# Supplementary material for: Crystal structures of human MGST2 reveal synchronized conformational changes regulating catalysis
Source: Nat Commun. 2021 Mar 19;12:1728. doi: 10.1038/s41467-021-21924-8 (PMC7979937; doi:10.1038/s41467-021-21924-8)
Supplement: Supplementary file 7 — Reporting Summary [file 41467_2021_21924_MOESM7_ESM.pdf]

Corresponding author(s): Jesper Z Haeggström

Last updated by author(s): Feb 9, 2021

Reporting Summary

Nature Research wishes to improve the reproducibility of the work that we publish. This form provides structure for consistency and transparency in reporting. For further information on Nature Research policies, see our Editorial Policies and the Editorial Policy Checklist.

Statistics

For all statistical analyses, confirm that the following items are present in the figure legend, table legend, main text, or Methods section.

n/a	Confirmed	
☐	☒	The exact sample size (*n*) for each experimental group/condition, given as a discrete number and unit of measurement
☐	☒	A statement on whether measurements were taken from distinct samples or whether the same sample was measured repeatedly
☒	☐	The statistical test(s) used AND whether they are one- or two-sided<br>*Only common tests should be described solely by name; describe more complex techniques in the Methods section.*
☒	☐	A description of all covariates tested
☒	☐	A description of any assumptions or corrections, such as tests of normality and adjustment for multiple comparisons
☐	☒	A full description of the statistical parameters including central tendency (e.g. means) or other basic estimates (e.g. regression coefficient) AND variation (e.g. standard deviation) or associated estimates of uncertainty (e.g. confidence intervals)
☒	☐	For null hypothesis testing, the test statistic (e.g. *F*, *t*, *r*) with confidence intervals, effect sizes, degrees of freedom and *P* value noted<br>*Give P values as exact values whenever suitable.*
☒	☐	For Bayesian analysis, information on the choice of priors and Markov chain Monte Carlo settings
☒	☐	For hierarchical and complex designs, identification of the appropriate level for tests and full reporting of outcomes
☒	☐	Estimates of effect sizes (e.g. Cohen's *d*, Pearson's *r*), indicating how they were calculated

Our web collection on statistics for biologists contains articles on many of the points above.

Software and code

Policy information about availability of computer code

Data collection	X-ray data collections were done using GDA at Diamond Light Source and MxCuBE at European Synchrotron Radiation Facility.<br>Phosphor imaging was done using Image Reader 8.0J/Image Gauge.
Data analysis	XDS Program Package Version 2: Diffraction data reduction<br>CHAINSAW and Refmac5 in CCP4 suit version 7.0 : Crystallographic software suit for x-ray data processing and refinement<br>PHASER in Phenix versions 1.13-2998 and 1.9-1683: Crystallographic software suit for molecular replacement and refinement<br>COOT Version 0.8.9.2: Protein model building<br>GROMACS version 5.1.4: Molecular simulations<br>PyMOL 2.3.2 and VMD 1.9.3: Molecular graphics softwares<br>Graphpad Prism 8.0: Data analysis

For manuscripts utilizing custom algorithms or software that are central to the research but not yet described in published literature, software must be made available to editors and reviewers. We strongly encourage code deposition in a community repository (e.g. GitHub). See the Nature Research guidelines for submitting code & software for further information.

Data

Policy information about availability of data

All manuscripts must include a data availability statement. This statement should provide the following information, where applicable:

- Accession codes, unique identifiers, or web links for publicly available datasets
- A list of figures that have associated raw data
- A description of any restrictions on data availability

Protein coordinates and structure factors of MGST2 are submitted to Protein Data Bank with accession codes 6SSR, 6SSS, 6SSU, 6SSW.
The PDB IDs 2UUI and 2PNO used in this study are available in Protein Data Bank.
MD trajectories and analysis scripts are available upon request.

Field-specific reporting

Please select the one below that is the best fit for your research. If you are not sure, read the appropriate sections before making your selection.

☒ Life sciences ☐ Behavioural & social sciences ☐ Ecological, evolutionary & environmental sciences

For a reference copy of the document with all sections, see nature.com/documents/nr-reporting-summary-flat.pdf

Life sciences study design

All studies must disclose on these points even when the disclosure is negative.

Sample size	Individual mutants were assayed for LTC4 synthase activity in three to four independent activity measurements.<br>MD simulations were done in triplicates.
Data exclusions	No data was excluded
Replication	Crystallization has been successfully repeated at least five times with reproducible crystals.<br>LTC4 synthase activity, UV difference spectrum measurements are reproducible for three independent measurements. Similar band pattern was observed in three independent trypsin digestions.
Randomization	Animals or human research participants were not involved in this study, and, as such samples were not randomized for the experiments.
Blinding	Blinding is not applicable to this study because data are derived from biophysical and biochemical methods with minimal risk of bias.

Reporting for specific materials, systems and methods

We require information from authors about some types of materials, experimental systems and methods used in many studies. Here, indicate whether each material, system or method listed is relevant to your study. If you are not sure if a list item applies to your research, read the appropriate section before selecting a response.

Materials & experimental systems

n/a	Involved in the study	
☒	☐	Antibodies
☒	☐	Eukaryotic cell lines
☒	☐	Palaeontology and archaeology
☒	☐	Animals and other organisms
☒	☐	Human research participants
☒	☐	Clinical data
☒	☐	Dual use research of concern

Methods

n/a	Involved in the study	
☒	☐	ChIP-seq
☒	☐	Flow cytometry
☒	☐	MRI-based neuroimaging
